# Modulation of Magnetic Properties at the Nanometer Scale in Continuously Graded Ferromagnets

**DOI:** 10.3390/ma11020251

**Published:** 2018-02-06

**Authors:** Lorenzo Fallarino, Patricia Riego, Brian J. Kirby, Casey W. Miller, Andreas Berger

**Affiliations:** 1 CIC nanoGUNE, Tolosa Hiribidea 76, E-20018 Donostia-San Sebastian, Spain; l.fallarino@hzdr.de (L.F.); p.riego@nanogune.eu (P.R.); 2 Helmholtz-Zentrum Dresden-Rossendorf, Institute of Ion Beam Physics and Materials Research, Bautzner Landstrasse 400, 01328 Dresden, Germany; 3 Departamento de Física de la Materia Condensada, Universidad del País Vasco, UPV/EHU, E-48080 Bilbao, Spain; 4 NIST Center for Neutron Research, NIST, Gaithersburg, MD 20899, USA; brian.kirby@nist.gov; 5 School of Chemistry and Materials Science, Rochester Institute of Technology, Rochester, NY 14623, USA; cmilleratphysics@gmail.com

**Keywords:** graded materials, magnetic films, magnetic multilayers, designed magnetic properties, magnetization reversal

## Abstract

Ferromagnetic alloy materials with designed composition depth profiles provide an efficient route for the control of magnetism at the nanometer length scale. In this regard, cobalt-chromium and cobalt-ruthenium alloys constitute powerful model systems. They exhibit easy-to-tune magnetic properties such as saturation magnetization *M_S_* and Curie temperature *T_C_* while preserving their crystalline structure over a wide composition range. In order to demonstrate this materials design potential, we have grown a series of graded Co_1−*x*_Cr*_x_* and Co_1−*w*_Ru*_w_* (10$\bar{1}$0) epitaxial thin films, with *x* and *w* following predefined concentration profiles. Structural analysis measurements verify the epitaxial nature and crystallographic quality of our entire sample sets, which were designed to exhibit in-plane *c*-axis orientation and thus a magnetic in-plane easy axis to achieve suppression of magnetostatic domain generation. Temperature and field-dependent magnetic depth profiles have been measured by means of polarized neutron reflectometry. In both investigated structures, *T_C_* and *M_S_* are found to vary as a function of depth in accordance with the predefined compositional depth profiles. Our Co_1−*w*_Ru*_w_* sample structures, which exhibit very steep material gradients, allow us to determine the localization limit for compositionally graded materials, which we find to be of the order of 1 nm. The Co_1−*x*_Cr*_x_* systems show the expected U-shaped *T_C_* and *M_S_* depth profiles, for which these specific samples were designed. The corresponding temperature dependent magnetization profile is then utilized to control the coupling along the film depth, which even allows for a sharp onset of decoupling of top and bottom sample parts at elevated temperatures.

## 1. Introduction to Graded Materials

**Figure 1:**
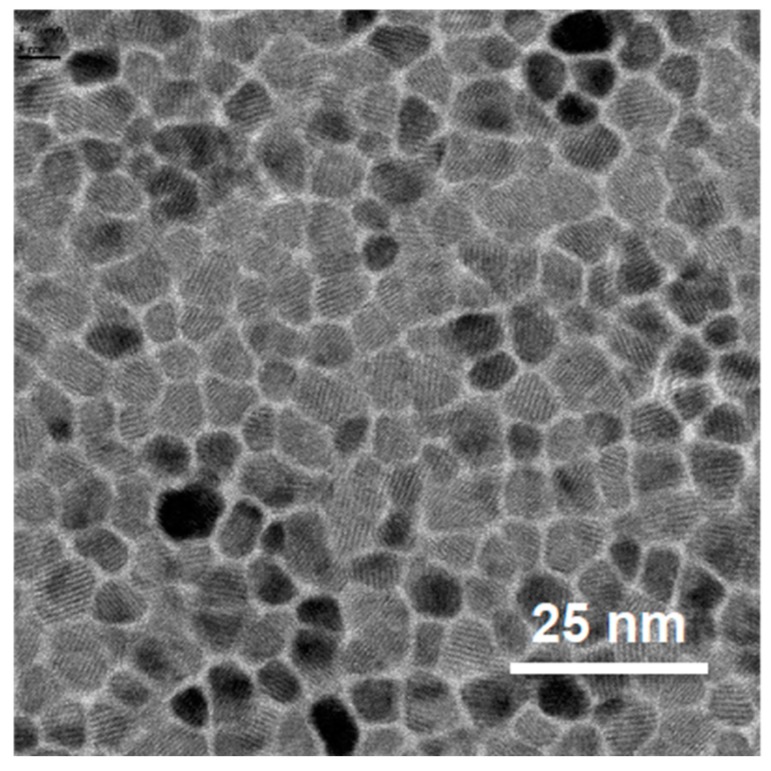
Top-view transmission electron microscopy picture of the nanoscale granular structure of modern magnetic recording media for hard disk drive applications. Reprinted with permission from [10]. Copyright 2011, Elsevier B.V.

In recent years, graded magnetic materials exhibiting spatially dependent magnetic properties have developed into a very relevant research area as top-down nanoscale materials fabrication techniques have become increasingly available [1,2,3,4,5,6,7]. Hereby, it is interesting to realize that fabrication of materials with spatially modulated magnetic properties for application purposes is actually not a very novel concept, but rather a well-known and well-utilized approach. Originally, however, spatial non-uniformities were generated solely via self-assembly or segregation dynamics rather than by means of top-down direct materials deposition control. Common examples of such graded materials are hard-magnetic alloys [8] or magnetic recording media [9,10]. Notably, the latter example of recording media for hard disk drive (HDD) applications has been utilized in the realm of nanoscale laterally graded materials for decades. This can be seen in Figure 1, which shows a top view transmission electron microscopy image of a nearly decade old magnetic recording media sample [10]. Here, the dark-appearing nanoscale ferromagnetic grains are separated by a light color grain boundary material that has a higher concentration of a non-magnetic segregant compound, commonly an oxide material, and thus is not itself ferromagnetic [10]. Correspondingly, the compositional structure along each lateral grain/boundary layer/grain sequence is sharply graded and exhibits a maximum segregant composition within the boundary layer that suppresses (or massively reduces) the exchange coupling between neighboring ferromagnetic grains [9,10]. This in turn allows for the individual magnetic state modification of each grain, thus leading to the very high magnetic information densities and capacities that HDD technology has achieved [9]. Such nanoscale magnetic design is enabled by compositionally graded materials that can be generated cost effectively by appropriate layer and processing control and was made possible by decades of materials processing and deposition research work.

A qualitative step forward from this growth-induced graded materials design has become possible more recently via the growth of materials that exhibit (sub) nm-scale depth-depend profiles [4,5,6,7,11,12,13,14,15,16,17]. The key difference here is that one can create exact growth profiles on the nm-scale along the depth direction, whereas segregation dynamics only provides a level of control as far as statistical averages are concerned. Originally, the depth-dependent grading concept was utilized in magnetic structures that consisted of very abrupt layering, such as the ferromagnetic/non-magnetic/ferromagnetic layer systems, in which giant magneto-resistance (GMR) was discovered and most successfully applied in magnetic read heads [18,19]. Other materials that utilize a layered fabrication process, e.g., Co/Pt-multilayers, should probably not be considered as graded materials, but rather nanoscale designed meta-materials because they exhibit rather uniform magnetic properties without depth-dependent magnetization states and they are not significantly different from equivalent homogeneous magnetic materials [20]. Another example of a graded material with nanoscale depth design are metamagnetic/ferromagnetic structures that can exhibit a very steep magnetic coercivity *H_c_* vs. temperature *T* dependence, making them a prime candidate for thermally assisted magnetic recording [21]. Despite their promise, these structures have yet to achieve technological relevance, and may soon have to compete with magnetic materials whose naturally occurring depth-dependent FM/AFM layer structure leads to extremely large *H_c_* vs. *T* dependencies [22,23].

More recently, anisotropy graded materials have become relevant in recording media for HDD applications [7,24,25]. Here, a pre-designed graded anisotropy depth structure of each grain is utilized to achieve an in-field magnetization reversal state that is non-collinear in nature. Thus, the relevant magnetic energy landscape for in-field reversal is different than that for pure thermal magnetic switching, allowing a media design, for which writability and thermal stability can be separately optimized. This approach bypassed a major technological bottleneck [9]. As such, anisotropy graded materials, also known as exchange spring media (after their non-collinear spring-like magnetic state during reversal), are a recent technologically relevant example, for which a very specific smoothly graded structure was proposed and utilized.

## 2. Prior Work on Exchange Graded Layer Structures

In a recently reported work, it was demonstrated that not only anisotropy gradients can be realized and tailored in compositionally graded films, but that it is also possible to engineer thermodynamically induced phase boundaries parallel to the sample surface by means of materials tuning [26]. In fact, a vertically graded Ni*_x_*Cu_1−*x*_ alloy sample structure was used to demonstrate the ability to map the temperature-dependent magnetic order dependency onto a depth-dependent spatial profile, which could furthermore be altered by temperature or magnetic field. Fundamentally, it is important to note that a magnetic material that exhibits ferromagnetic exchange coupling in its entirety undergoes only one ferromagnetic phase transition at one single Curie temperature *T_C_*. Practically, however, material systems with compositional gradients can be thought of as having a continuous distribution of “local” or effective *T_C_* values, which is the origin of the above mentioned mapping of a magnetization *M* vs. *T* curve into a spatial profile as demonstrated in [26]. The fact that such depth-dependent *T_C_* modulations were found to occur even at the nm length scale is a very relevant result, because it is the very length scale that is being utilized in nanotechnology applications today, such as HDD media structures.

This surprisingly strong localization of magnetic behavior in ferromagnetic exchange graded materials [26] is the motivation of our present work. We review this subject and extend on our recent studies [27,28], for which we utilized high-quality epitaxial films to fabricate “textbook” cases of magnetic layer structures with compositionally graded profiles. This approach allowed for a detailed evaluation of the underlying physics of magnetization states that are found in such systems, as demonstrated in this work. 

In particular, we address the question of the minimum length-scale, at which the local “*T_C_*” picture is still a viable concept. This physical picture has to break down at some point because the materials we are investigating here are metallic ferromagnets, so that the delocalized nature of the itinerant ferromagnetism must become relevant at some length scale, making a truly local description impossible. Moreover, ferromagnetism is fundamentally a collective phenomenon, and as such local representation of its properties must fail at some length scale, even in cases in which its origin, i.e., the exchange interaction, is extremely localized. 

In addition, our work aims to explore whether and how this unexpectedly localized behavior could affect the collective magnetization reversal of appropriately designed graded structures. The main purpose is the identification of structures and strategies, for which unusual or anomalous temperature dependent changes of reversal can be generated that would not occur in otherwise similar but homogenous material systems. Such non-trivial collective magnetization reversal behavior might then be utilized in novel device concepts for a variety of possible applications. 

## 3. Epitaxial Graded Structures

### 3.1. Test Structures 

In order to perform a detailed and quantitative analysis of magnetic properties in compositionally graded structures, it is convenient to select a specific thin film material system that can be grown epitaxially with uniaxial magnetocrystalline anisotropy in the plane of the film to achieve negligible in-plane demagnetization fields. Correspondingly, the system develops a uniform magnetization state, which is restricted only to in-plane orientations. Therefore, in spite of complex compositional gradient structures, magnetization properties and related magnetization reversal should be simple enough to allow for a comprehensive and precise quantitative data analysis by using a basic macrospin model [29,30]. Among the elemental ferromagnets, bulk Co adopts the hexagonal close packed (*hcp*) crystal structure at room temperature and exhibits a magnetic easy axis (EA) behavior along the *c*-axis. Thus, we have utilized Co and Co-based materials for our films, which can be grown epitaxially with the necessary in-plane *c*-axis orientation [27,30]. 

**Figure 2:**
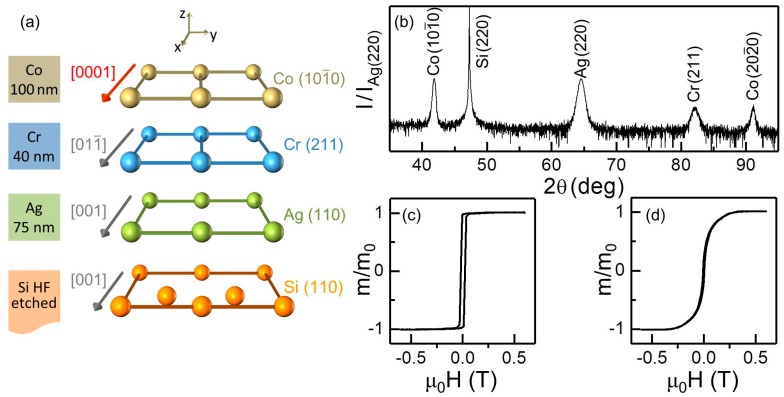
(Color online) (**a**) schematic of the crystallographic orientation relationships between Si (110), Ag (110), Cr (211) and Co (10$\bar{1}$0) lattices; (**b**) XRD θ/2θ scan of a 100 nm thick Co reference sample grown on a Cr (40 nm)/Ag (75 nm) bilayer; (**c**,**d**) VSM measurements with the externally applied magnetic field parallel to the easy (**c**) and hard (**d**) axes.

Figure 2a shows the specific layer sequence that we used, including all template layers, and the epitaxial relationships between them. As substrates, Si (110) oriented wafers have been utilized, on top of which 75 nm thick Ag and 40 nm thick Cr bilayers have been deposited to promote the desired crystallographic orientation of the Co layers having an in-plane EA. Figure 2b shows a typical θ–2θ X-ray diffraction (XRD) measurement for one of our homogeneous epitaxial base structures, namely a 100 nm thick Co film. Only the Si (220), Ag (220), Cr (211), Co (10$\bar{1}$0) and Co (20$\bar{2}$0) diffraction peaks are present, corroborating the epitaxial nature of the sample. For Co, apart from a narrow first order (10$\bar{1}$0) diffracted signal, the second order (20$\bar{2}$0) diffracted peak is also measured, which in itself is a relevant experimental evidence of the good crystallographic quality of our magnetic films. Figure 2c,d show room temperature easy and hard (HA) axes magnetization curves for a 100 nm thick Co sample. Along the EA, Figure 2c, the measurement shows a perfectly square-shaped hysteresis with full remanence and an abrupt change of magnetization during the reversal. On the contrary, Figure 2d does not show any switching during the reversal process and accordingly no hysteretic behavior, which indeed corresponds to a completely reversible change in the magnetization orientation, typical of HA orientations. Both measurements verify the intended uniaxial magnetic anisotropy of the Co-film.

It is beneficial to point out here that this type of epitaxial structure is just a “building block” sub-structure for our study. For compositionally graded materials, we have exploited the possibility of alloying Co with Cr or Ru in order to modify and tune key magnetic properties such as saturation magnetization (*M_S_*) and Curie temperature *T_C_*. Cobalt-chromium (Co_1−*x*_Cr*_x_*) and cobalt-ruthenium (Co_1−*w*_Ru*_w_*) alloys are both most suitable model systems in this regard, as they are ferromagnetic up to *x* = 0.35 and *w* = 0.36 [30,31,32], with magnetic properties that are easily tunable by changing the dopant concentration *x* or *w*. It is also of crucial importance that these alloys maintain the lattice structure and form stable solid solutions in a wide range of dopant concentration so that epitaxial growth can be maintained even in the presence of depth-dependent compositional gradients. In the following section, the specific compositional depth profiles that were explored and studied will be presented and discussed.

### 3.2. Explored Depth Profiles

**Figure 3:**
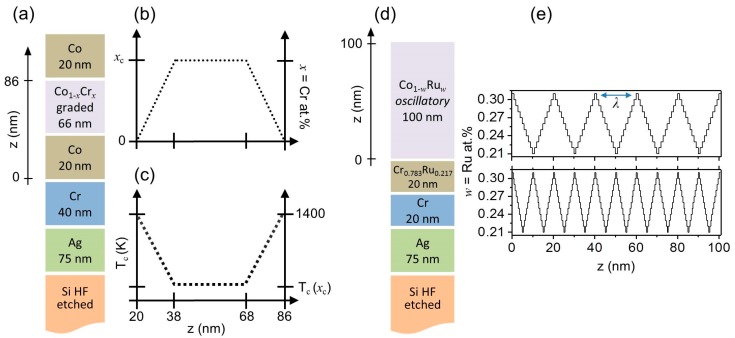
(Color online) (**a**) schematic of the layer growth sequence for the bathtub-like sample studied in this work; (**b**) shows the corresponding Cr content and (**c**) the associated local *T_C_* depth profiles. *T* = 1400 K corresponds to the Curie temperature of pure cobalt; (**d**) schematic of the layer growth sequence for the oscillatory-like sample studied in this work; (**e**) shows the Ru modulations for the *λ* = 20 nm (top) and *λ* = 10 nm (bottom) samples.

The extension of magnetically graded heterostructures, such as the one studied in reference [26], to systems with rationally designed composition profiles was initiated in reference [27]. The main motivation was to achieve desired temperature and magnetic field dependencies in order to trigger complex collective or non-collective magnetic behavior dictated solely by compositional gradients [27]. The first specific test case was a symmetrically graded structure with a low local Curie temperature in its center exhibiting an overall bathtub-like profile. Such a structure has shown a very distinct temperature-dependent magnetic coupling along its thickness, since the ferromagnetic region at the center of the structure (low *T_C_*) loses its ferromagnetic state at a lower temperature than the adjacent material. A series of symmetrically graded CoCr samples were fabricated with the maximum dopant concentration in the center (hereinafter referred to as *x*_c_) ranging from *x*_c_ = 0.25 to 0.32. Figure 3a shows a schematic of the selected layer growth sequence. A series of vertically mirrored and graded Co/Co_1−*x*_Cr*_x_*/Co films have been grown on top of a Si/Ag/Cr layers sequence. Figure 3b, referring to the central 66 nm thick graded Co_1−*x*_Cr*_x_* layer, displays its Cr content depth profile while Figure 3c illustrates the expected corresponding local *T_C_* distribution derived from the combination of the nearly linear relationship between *T_C_* and *x* and the compositional depth profile itself [30,31,32]. It is worth mentioning that the central Co_1−*x*_Cr*_x_* layer, as shown in Figure 3b,c, indeed consists of three sub-regions: a first 18 nm thick part, in which the content of Cr increases linearly from *x* = 0 to its maximum value *x*_c_; a 30 nm thick central region, in which *x* is constant; and another 18 nm thick layer, in which *x* decreases from *x*_c_ down to 0. The result is a magnetic structure that is nominally symmetric with respect to its center. This structure allowed for a temperature dependent tuning of the depth-dependent correlation of magnetic states and reversal behavior. 

While reference [26] demonstrated that the local Curie temperature of a graded structure can be continuously varied via compositional gradients over tens of nanometers, an itinerant ferromagnetic system should not exhibit purely local properties and the exchange coupling along the *z*-axis should start to dominate the effects of any compositional gradient over short distances. Understanding this localization limit is important for potential applications, as it dictates the length scale, below which graded materials design ceases to be a viable engineering strategy. In order to study this limit of local *T_C_*-depth profile generation via compositionally graded materials, we have explored a more complicated structure, specifically an oscillatory compositional depth profile produced by a “triangular” Ru content profile using different oscillation periods *λ*. By changing the *T_C_*-oscillation period we aimed to explore in a systematic and elegant way the lower limit of the ability to transfer compositional effects into modulated magnetic states. In our samples, the Ru concentration *w* was linearly decreased from 0.31 to 0.21, and subsequently increased linearly back to 0.31 in each period. A series of these “triangular” graded Co_1−*w*_Ru*_w_* epitaxial thin films were grown on top of a slightly modified template layer sequence (discussed in the following section) Si/Ag/Cr/CrRu and with *λ* = 10 and 20 nm. Figure 3d shows a schematic of the layer growth sequence, while Figure 3e shows the corresponding Ru content depth profile. CoRu was chosen for the high gradient structures instead of CoCr because Ru presents contrast advantages for subsequent Polarized Neutron Reflectometry (PNR) measurements, as the nuclear scattering length of Ru is nearly twice that of Cr and thrice that of Co [33], leading to high sensitivity measurements and thus allowing for the corroboration of the actual sample gradient structure. 

### 3.3. Experimental Characterization Methods

It is worthwhile to briefly mention the experimental systems utilized in the present work. The structural analysis of our samples was performed by means of X-ray diffraction (XRD) utilizing a X’Pert Pro diffractometer (PANalytical) with Cu *K_α_* radiation. Room temperature magnetic characterization was performed using a commercial MicroMag 3900 vibrating sample magnetometer (VSM) from Princeton Measurements Corporation, equipped with an automated 360° rotational stage that allows for the rotation of the sample around the axis perpendicular to the applied field, with an angular precision of better than 1°. Temperature dependent magnetic measurements were performed using a commercial MPMS3 SQUID-VSM magnetometer (Quantum Design). Magneto-optical Kerr effect (MOKE) data were measured by means of an experimental setup in the longitudinal MOKE configuration at a laser wavelength of *λ* = 635 nm [34,35]. The polarized neutron reflectometry measurements were carried out on the PBR beamline at the NIST Center for Neutron Research. By using a Fe/Si supermirror/Al-coil assembly, a monochromatic *λ* = 0.475 nm neutron beam was spin polarized either parallel (+) or antiparallel (−) to the external applied magnetic field H, which was always applied parallel to the magnetic easy axis. The beam was specularly reflected by the sample surface and measured as a function of wave-vector transfer along the surface normal *Q* using a ^3^He detector. The data were corrected for background, beam polarization and beam footprint. Specular non-spin-flip (*R*^−^^−^,*R*^++^) and spin-flip (*R*^+^^−^,*R*^−^^+^) reflectivities were measured under various field and temperature conditions [36]. The first and second superscripts denote the spin orientation of the incoming and scattered neutrons, respectively. In our experiments, only very weak spin-flip scattering was detected, implying that the magnetization stays essentially collinear with the magnetic field. We have therefore focused our investigation only on the non-spin-flip measurements. Model fitting was performed using the NIST Refl1D software package [37] with parameter uncertainties determined using a Markov chain Monte Carlo algorithm [38].

### 3.4. Fabrication and Epitaxial Properties

All samples have been fabricated by means of sputter deposition utilizing a UHV system (ATC series by AJA International). Prior to any deposition, the Si (110) oriented substrates were cleaned in sequence by acetone, isopropanol and deionized water. The native amorphous silicon oxide was then etched by using a wet hydrofluoric acid (HF) chemical bath, after which the substrates were immediately transferred into the UHV sputter system. All depositions were performed at room temperature (RT) using a pure Ar pressure of 4 × 10^−1^ Pa. It is worth mentioning that our deposition temperature was specifically selected in order to avoid diffusion or segregation effects that commonly occur at elevated temperatures [39,40,41,42,43]. The symmetric (oscillatory) layer structures were deposited by co-sputtering of Co and Cr (Ru) targets keeping the power of Co constant while changing that of the dopant material in order to achieve the intended composition profile. The initial onset layer of 75 nm thick Ag is common in both structures, while the subsequent template layer sequence differs for the two types of Co alloys that we used. 

A 40 nm thick Cr layer was deposited prior to the growth of the symmetric CoCr graded film structure shown in Figure 3a–c. This is necessary to achieve a high quality 20 nm thick (10$\bar{1}$0) oriented Co layer [30,44], which in turn served as a template for the epitaxial growth of the 66 nm thick compositionally graded Co_1−*x*_Cr*_x_* layer. On top of this graded layer, another 20 nm thick (10$\bar{1}$0) oriented Co layer was grown and subsequently covered by an amorphous 10 nm Si-oxide layer for surface protection.

In the case of the oscillatory graded CoRu structure, the above described layer sequence had to be altered as was already known from our prior work on uniform Co_1−*w*_Ru*_w_* alloy layer films [30]. Specifically, it was necessary to increase the lattice constant of the Cr template layer by alloying it with Ru [30,45] in order to improve the epitaxial growth of the magnetic Co_1−*w*_Ru*_w_* layers. We thus added a 20 nm thick Cr_1−*y*_Ru*_y_* intermediate layer on top of a 20 nm thick Cr underlayer, as shown in Figure 3d. The optimal stoichiometry for the Cr_1−*y*_Ru*_y_* has been selected following reference [30], where it was found that a ratio of *w*/*y* = 1.2 in between the two Ru alloy contents leads to consistently excellent growth conditions. It is worth mentioning that in this work the average Ru content of the magnetic layer $\bar{W}$ has been taken into account in order to calculate the optimal *y* Ru concentration. The magnetic 100 nm thick CoRu films were then grown by continuously varying the dopant content following a triangular waveform along the growth direction. The result was a magnetic structure that was nominally symmetric with respect to the center of each oscillation period *λ*. As a final deposition step, each sample was coated with 10 nm of amorphous Si-oxide in order to avoid any oxidation or contamination.

As relevant reference structures, three additional samples were fabricated: an epitaxial 106 nm thick pure Co sample (*x*_c_ = 0), which was deposited using the same underlayer sequence as for the symmetrically graded CoCr structure; and two 100 nm thick uniform CoRu alloy films with composition Co_0.69_Ru_0.31_ and Co_0.79_Ru_0.21_, which utilized the appropriate CrRu containing underlayer sequence. Also the reference samples were coated with 10 nm thick amorphous Si-oxide for environmental protection. It is worth remarking that the intended preservation of the original *hcp* crystal structure of pure Co and of the in plane alignment of the *c*-axis, which coincides with the magnetic easy axis, allow for a comprehensive and precise quantitative data analysis by means of a rather simple macrospin model [30]. Accordingly, we conducted thorough structural measurements of all our samples to verify their epitaxial quality and in-plane *c*-axis orientation over the entirety of the explored compositional ranges and gradient structures. 

**Figure 4:**
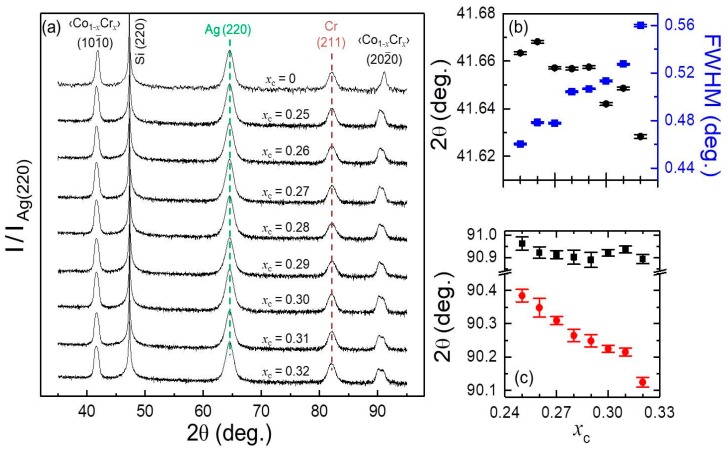
(Color online) (**a**) XRD θ–2θ measurements for “bathtub-like” samples with different Cr concentrations *x*_c_. Each scan has been normalized to the intensity of its Ag (220) peak; (**b**) XRD ‹Co_1−*x*_Cr*_x_*› (10$\bar{1}$0) peak positions (black dot) and FWHM (blue squares) as a function *x*_c_; (**c**) XRD ‹Co_1−*x*_Cr*_x_*› (20$\bar{2}$0) peak positions as a function *x*_c_ evaluated by a double Gaussian fit function.

Figure 4a shows XRD θ–2θ scans for symmetrically graded samples that were grown with different maximum Cr concentrations *x*_c_ at the center of the magnetic structure. The absence of any diffraction peak other than Si (220), Ag (220), Cr (211), ‹Co_1−*x*_Cr*_x_*› (10$\bar{1}$0) and (20$\bar{2}$0) (the simplified nomenclature ‹Co_1-_*_x_*Cr*_x_*› will be used for the remainder of the XRD characterization, representing the total 106 nm thick film structure) along with the fact that higher order diffraction signals are clearly visible for the magnetic film in all cases, indicate that the crystallographic quality of our symmetrically graded samples is excellent. Furthermore, the Ag and Cr template layer peaks look virtually the same for all samples, both in terms of angular position and full width at half-maximum peak intensity (FWHM), verifying the robustness of our fabrication process. This template layer stability allows us to ascribe any significant change in the graded layer XRD peaks to the specific Cr graded layer growth itself. Most relevantly, the entire set of symmetrically graded samples exhibits a crystallographic orientation quality that is very similar to the pure Co reference sample (*x*_c_ = 0) despite the complex depth-dependent structure. Nonetheless, there are differences that need to be considered. For instance, the nature of the graded magnetic layer peak is different from the usual x-ray diffraction peaks in homogeneous systems. Here, each of the Bragg peaks corresponding to the ‹Co_1−*x*_Cr*_x_*› (10$\bar{1}$0) and (20$\bar{2}$0) XRD signal is the result of a superposition of the signals generated by the two outer Co layers and the signal coming from the central graded structure. By means of a single Gaussian peak fitting function, the angular position of the (10$\bar{1}$0) peaks is found to shift towards lower diffraction angles with increasing *x*_c_ whereas the corresponding FWHM increases, as shown in Figure 4b as (black) dots and (blue) squares, respectively. Typically, such a peak width increase is related to a reduced quality of the epitaxial growth, but here it originates from a lattice expansion in the central part of the graded layer due to the substitution of Co by Cr atoms. This evolution is more visible for the (20$\bar{2}$0) second order Bragg reflections, for which an actual splitting of the XRD-peak is observed for the graded layer structures. By using a double Gaussian peak fitting function, the position of the (20$\bar{2}$0) peaks corresponding to the central region was found to decrease with increasing *x*_c_, whereas the diffraction angles corresponding to the outer parts of the graded layer structure stay constant in the entire concentration range investigated by us, as shown by the data in Figure 4c, which are displayed as (red) dots and (black) squares, respectively. 

**Figure 5:**
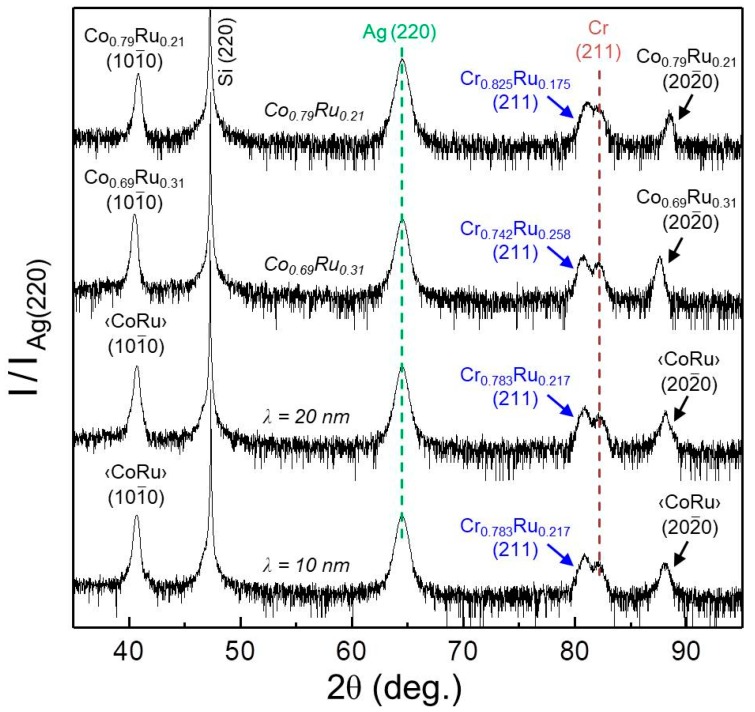
(Color online) XRD θ–2θ scans for samples with uniform *w* = 0.21 and *w* = 0.31, 20 nm modulated *w* = 0.21–0.31, and 10 nm modulated *w* = 0.21–0.31. Each scan has been normalized to the intensity of its Ag (220) peak.

A consistently high epitaxial quality has also been achieved for the oscillatory CoRu graded film structures. Figure 5 shows XRD θ–2θ scans in the angular range 35° ≤ 2θ ≤ 95° for two different wavelength samples (*λ* = 10 and 20 nm) in comparison with results for uniform *w* = 0.21 and *w* = 0.31 reference films. All the scans exhibit only the Si (220), Ag (220), Cr (211), Cr_1*−y*_Ru*_y_* (211), Co_1−*w*_Ru*_w_* (10$\bar{1}$0) and (20$\bar{2}$0) diffraction peaks, corroborating the epitaxial nature of the samples. Specifically the occurrence of the higher order (20$\bar{2}$0) XRD-peak for the magnetic CoRu structures is a sign of the good crystallographic quality of these films, including the oscillatory composition Co_1−*w*_Ru*_w_* films. We also see in Figure 5 that the diffracted peaks arising from the (211) oriented Cr_1*−y*_Ru*_y_* layers can be observed at lower angles than the diffracted signal from the (211) Cr layer. This shift to lower angles demonstrates the increase of the lattice constant of the Cr template layer by alloying it with Ru. In addition, the Co_1−*w*_Ru*_w_* *(n0$\bar{n}$0)* X-ray diffraction peaks are all very similar in their height and width, verifying the achievement of a quality for the oscillatory epitaxial films that is not compromised by their complex depth dependent structure. Indeed, a wavelength dependent evolution of the latter diffraction peaks can be observed in Figure 5. By reducing *λ*, the oscillation fringes that are caused by the multilayer nature of the structure, are shifting with respect to the central position of the peak.

### 3.5. Macroscopic Magnetic Properties

#### 3.5.1. Uniaxial Magnetic Anisotropy

**Figure 6:**
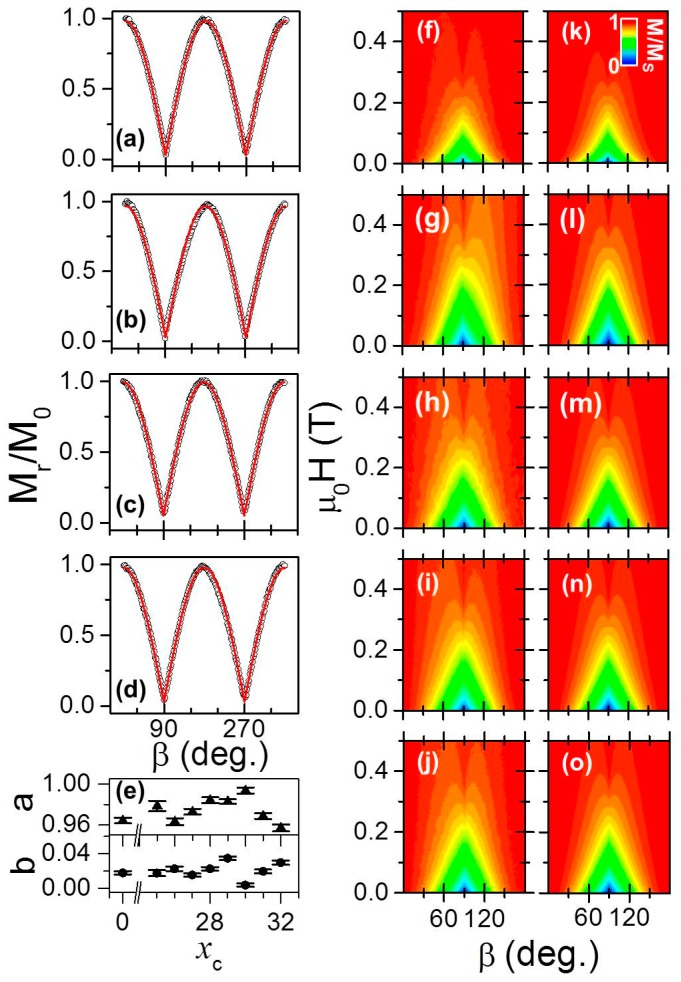
(Color online) (**a**–**d**) Measured *M_r_*/*M*_0_ values for four ‹Co_1−*x*_Cr*_x_*› samples with different Cr concentrations *x*_c_ = 0 (**a**); 0.25 (**b**); 0.28 (**c**); 0.30 (**d**) (opened black dots). The red lines display the least squares fit of the *M_r_*/*M*_0_ data by using Equation (1) [44]. (**e**) Cr concentration *x*_c_ dependence of the extracted *a* and *b* parameters by least squares fit using Equation (1); (**f**–**j**) in-plane angular dependence of the magnetization as a function of the applied field angle and strength for five samples with different Cr concentrations *x*_c_ = 0 (**f**), 0.25 (**g**), 0.28 (**h**), 0.30 (**i**) and 0.32 (**j**); the data are displayed as color-coded maps and are normalized to the saturation magnetization *M_S_* evaluated by least-squares fits using Equation (2); (**k**–**o**) show the corresponding least-squares fits of the data determined upon minimization of the total energy [30].

In order to verify that the designed symmetrically and oscillatory graded structures exhibit the intended uniaxial magnetocrystalline anisotropy with a magnetic easy axis in the plane of the films and parallel to the crystallographic *c*-axis, the macroscopic magnetic properties were investigated and analyzed. The first sample structure discussed here is the symmetrically graded one. Figure 6a–d show as open black dots the experimentally determined remanent magnetization *M_r_* values as a function of the magnetic field angle *β* for four different samples, representing different *x_c_* and thus different compositional depth profiles. The remanent magnetization value for each *β*, which is defined by the in-plane applied field direction with respect to the crystallographic *c*-axis, was measured by sweeping the magnetic field strength from positive saturation down to zero at every field orientation angle in between *β* = 0° to *β* = 360° using a step size of 2°. In each case, *M_r_* has been normalized to a reference magnetization value *M*_0_, which is measured at the maximum applied field strength *µ*_0_*H* = 0.6 T. It can be clearly observed that a strong angular dependence of *M_r_*/*M*_0_ for all samples exists with an angular periodicity of 180°, which is the signature of uniaxial symmetry. It can also be appreciated that in each data set, the normalized remanent magnetization is almost exactly 1 along the EA (*β* = 0°, 180°) and its value decreases in a monotonic way as one goes further away from the EA towards the HA (*β* = 90°, 270°), where *M_r_* vanishes as expected. In order to quantify the degree of uniaxial anisotropy, we have performed least-squares fits to the data by using the formula [44]:

(1) $$\frac{M_{r}}{M_{0}}=b+a\left|\cos\beta \right|$$

in which *b* and *a* are fit parameters that describe the degree of uniaxial alignment. For isotropic samples, *b* takes a finite value between 0 and 1 and *a* equals 0. In contrast, the two values correspond to *b* = 0 and *a* = 1 for a perfect uniaxial anisotropy system. Least-squares fits to Equation (1) have been performed for the experimental *M_r_*/*M*_0_ data of all symmetrically graded samples. Figure 6a–d show the fitting results as (red) solid lines in direct comparison to the experimental data. In each case, we find excellent agreement between the experimental data and the least-squares fits according to Equation (1). The extracted *a* and *b* values, together with the associated errors estimated from each of the least-squares fits, are plotted in Figure 6e as a function of *x*_c_. Despite some degree of variations in between the extracted parameters for our samples, all values are consistent with excellent uniaxial grain alignment. Moreover, the average values for $\bar{a}$ = 0.98 ± 0.02 and $\bar{b}$ = 0.01 ± 0.01 that we have determined from our experiments are consistent within their statistically estimated error with the values *a* = 1 and *b* = 0 for a perfect uniaxial anisotropy system.

**Figure 7:**
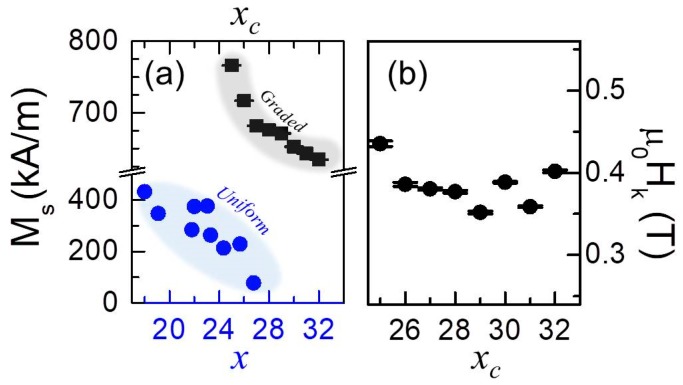
(Color online) (**a**) the bottom part shows, as (blue) dots, room temperature *M_S_* of uniform Co_1−*x*_Cr*_x_* alloy samples adapted from [31] plotted as a function of Cr content *x*; (**a**) top part displays, as (black) squares, extracted room temperature *M_S_* of symmetric graded epitaxial Co/Co_1−*x*_Cr*_x_*/Co (10$\bar{1}$0) samples as a function of *x*_c_. (**b**) Magnetocrystalline anisotropy fields *µ_0_H_K_* of symmetric graded epitaxial structures as a function of *x*_c_*.*

In general the determination of the magnetic anisotropy field $H_{k}=(H_{k_{1}}+H_{k_{2}})=({2k}_{1}/\mu_{0}M_{s}+{4k}_{2}/\mu_{0}M_{s})$ as well as the saturation magnetization *M_s_*, can be based on magnetization measurements performed just along the HA (i.e., *β* = 90° or 270°) [30]. In our work, however, the entire field orientation range has been utilized to determine the volume averaged magnetic properties in a very robust manner. Specifically, we acquired room temperature hysteresis loops for various orientations of the in-plane applied field. The results for samples with different *x*_c_ = 0, 0.25, 0.28, 0.30, 0.32 are depicted in Figure 6f–j as color-coded magnetization maps versus applied magnetic field strength *µ*_0_*H* and its angle of application *β.* The magnetization is plotted from positive saturation down to zero field at every field orientation angle from *β* = 0° to *β* = 180° using a step-size of 2° and it is normalized to *M*_S_. The data reveal that all samples show a marked uniaxial magnetic anisotropy with the *c*-axis being the preferential axis of magnetization. Indeed, near *β* = 90° (HA) a cone-shaped structure of reduced magnetization can be clearly observed due to the fact that insufficiently high external magnetic fields only generate negligible magnetization values along these field directions. In contrast, along *β* = 0°, 180° (EA) the external magnetic field has very little influence on the observed magnetization, because *M* is saturated along those directions even at remanence. In order to obtain a quantitative estimation of the macroscopic magnetic properties, least-squares fits of the entire (not normalized) data sets have been performed by using the macrospin model devised in reference [30], which is described by a total energy:

(2) $$E=-\mu_{0}M_{s}Hcos\left(\beta -\alpha \right)+k_{1}{sin}^{2}\alpha +k_{2}{sin}^{4}\alpha$$

where the saturation magnetization *M_S_* and the first- and second-order magnetocrystalline anisotropy constants, respectively *k*_1_ and *k*_2_ [30], are the only fit parameters. By means of energy minimization for each pair (*H*, *β*), the angle *α* defined by the direction of magnetization with respect to the easy axis is numerically determined [30]. Figure 6k–o show the results of these fits, which exhibit excellent agreement with the corresponding experimental data in Figure 6f–j, with an average R^2^-value of 0.975. The extracted values for the saturation magnetization *M*_S_ are shown in Figure 7a as a function of *x*_c_. Before going deeper into the analysis of the data, a related point to consider is the saturation magnetization of the pure reference *x*_c_ = 0 sample (not shown), which is very close to the known bulk value for cobalt, namely 1422 kA/m, verifying the good magnetic quality of the base structure [46]. 

As expected, in the case of the graded alloy films, *M_S_* decreases upon introducing Cr into the Co lattice and does so in a monotonic fashion with the Cr concentration *x*_c_. The reduction of *M*_S_ upon increasing *x*_c_ is not surprising, given that Cr atoms reduce the net ferromagnetic moment per atom. However, the graded systems remain ferromagnetic above *x* = 0.28, the value at which a uniform Co_1−*x*_Cr*_x_* alloy sample is expected to become paramagnetic at room temperature, as shown in Figure 7a [31,32]. To understand this, one needs to remember that within the same epitaxial film unit, the central graded structure is sandwiched in between two 20 nm thick pure Co layers, which are essentially not influenced by the compositional gradient. Correspondingly, it is evident that for the high *x*_c_ regime, the extracted *M_S_* values in Figure 7a do not drop down to zero due to the saturation magnetization of the two outer Co-layers. In fact, the total volume-averaged magnetization is constrained by the *M_S_* of the pure Co layers times their volume fraction, which is exactly what we observed experimentally. 

The room temperature anisotropy fields *H_k_* are plotted as a function of *x*_c_ in Figure 7b. An almost constant value of *H_k_* is observed, nearly independent of the graded film concentration. It is relevant to remark here that the reduction of *k*_1_ and *k*_2_ upon increasing *x*_c_ is very similar to the decrease of *M_S_* with *x*_c_, which implies a compensation of both effects leading to *H_k_* values being nearly constant in the entire concentration range. Thus, despite the complex depth-dependent structure of the samples, all of them exhibit a well-defined uniaxial magnetocrystalline anisotropy that is well understood. 

**Figure 8:**
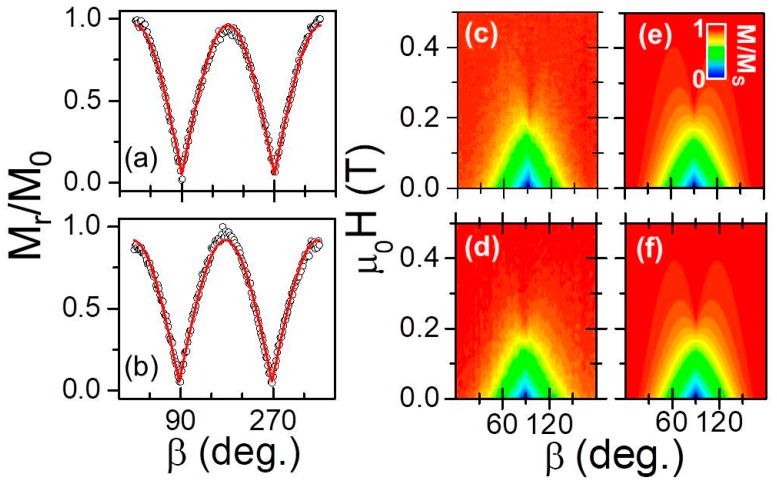
(Color online) (**a**,**b**) Measured *M_r_*/*M*_0_ values for the two oscillatory Co_1−*x*_Ru*_x_* samples with different *λ* = 10 (**a**), 20 (**b**) nm (opened black dots). The red lines show the least-squares fits of the *M_r_*/*M*_0_ data by using Equation (1). (**c**,**d**) In-plane angular dependence of the magnetization as a function of the applied field angle and strength for *λ* = 10 (**c**), 20 (**d**) nm. The data are displayed as color-coded maps and are normalized to the saturation magnetization *M_S_* evaluated by least-squares fits using Equation (2). (**e**,**f**) show the corresponding least-squares fits of the data determined upon minimization of the total energy [30].

We utilized the above-described methodology to verify that the oscillatory graded structures exhibit uniaxial magnetocrystalline anisotropy with an in-plane magnetic easy axis parallel to the *c*-axis. Figure 8a,b show as open black dots the experimentally determined remanent magnetization *M_r_* values as a function of the angle *β* for the two different wavelengths *λ* = 10 (a) and 20 (b) nm. Just as for the data shown in Figure 6a–d, *M_r_* has been normalized to a reference magnetization value *M*_0_ measured at the maximum applied field *µ*_0_*H* = 0.6 T. Again, we find a periodicity of 180° for *M_r_*/*M*_0_, a normalized magnetization of almost one along the EA (*β* = 0°) and nearly 0 for the HA (*β* = 90°), verifying uniaxial magneto-crystalline anisotropy. Figure 8a,b show the least-squares fitting results to Equation (1) as (red) solid lines in direct comparison to the experimental data, and which exhibit excellent agreement. The extracted *a* and *b* values, together with the associated errors estimated from each of the least-squares fits, are listed in Table 1. Both are consistent with values expected for uniaxial anisotropic samples. However, one can also appreciate that both values show relevant deviations from the perfect case, which can be attributed to a number of possible reasons and might be related to the real nature of these samples. Due to their oscillatory composition, a significant fraction of the sample volume is near its “local” Curie temperature during our room temperature magnetometry measurements. Thus, it is likely that these samples will exhibit a non-vanishing susceptibility even if aligned along the easy axis, which in turn leads to a reduction of the maximum remanent magnetization and correspondingly the extracted *a* values. This is exactly what we observe experimentally. 

Figure 8c,d display as color-coded maps our measured room temperature normalized magnetization *M/M_S_* data as a function of the magnetic field strength *µ*_0_*H* and the field angle *β*. These data sets were assembled as described above. Both experimental maps indicate that the samples exhibit uniaxial in-plane anisotropy with the easy axis coinciding with the *c*-axis, marked by the presence of a cone shaped structure of reduce magnetization near *β* = 90° (HA) and full remanence along *β* = 0° and *β* = 180° (EA). Thus, these data sets reveal that the anisotropy symmetry and the easy axis orientation are neither impacted by the Ru alloying, nor by the oscillatory compositional gradient structure. By performing least-squares fits of the entire (not normalized) data sets to Equation (2), the volume-averaged magnetic properties, specifically *H_k_* and *M_S_*, were extracted as a function of *λ*. Results of the fits are shown as color-coded maps in Figure 8e,f. They exhibit excellent agreement with the experimental data in Figure 8c,d with an average R^2^-value of 0.97. The room temperature saturation magnetization *M_S_* and anisotropy field *H_k_* values, extracted from the fits, are listed in Table 1. Both quantities stay constant upon changing the modulation period, and their values are in agreement with those of a uniform Co_1−*x*_Ru*_x_* alloy sample with *x* = 0.26, which is the average Ru content in our samples [30]. Therefore, all of our complex samples exhibit a well-defined, robust and simple uniaxial magnetic anisotropy, which will aid the interpretation of other experimental data, in particular our PNR measurement results.

#### 3.5.2. Magnetization Reversal in Symmetric Graded Structures

**Figure 9:**
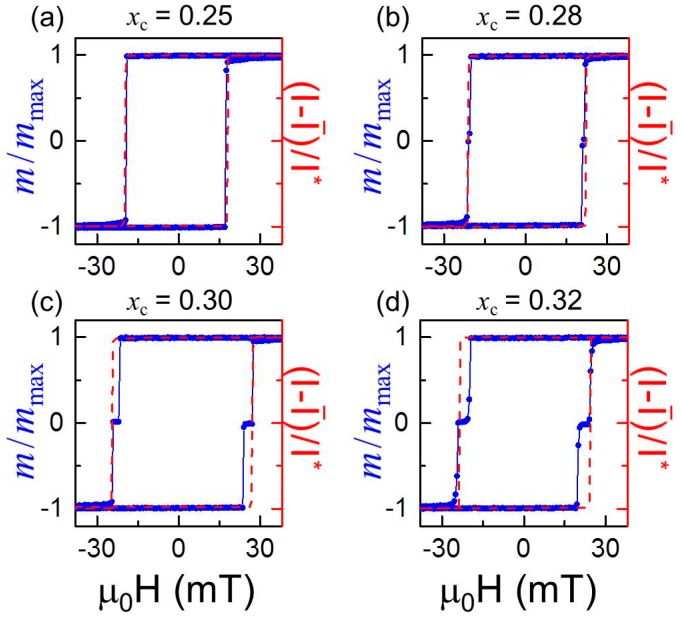
(Color online) VSM room temperature hysteresis loops (black dots) and corresponding MOKE measurements (red dashed lines) along the easy axes for four different samples with Cr concentrations *x*_c_ = 0.25 (**a**), 0.28 (**b**), 0.30 (**c**), 0.32 (**d**). Hereby, the VSM data are normalized to its maximum value *m*_max_ in each case, whereas the MOKE measurements are processed in each case by first subtracting = (*I*_max_ + *I*_min_)/2 and then the resulting difference has been normalized by I* = (*I*_max_ − *I*_min_)/2.

The characteristics of ferromagnetic materials are most frequently given in terms of their isothermal hysteresis loop. Figure 9a–d show VSM room temperature M(H) measurements, plotted as (black) dots, for four selected samples measured with the external magnetic field applied parallel to the easy axis. This particular orientation has been selected due to absence of any magnetization rotation process during its reversal. Figure 9a displays data for the sample with the lowest explored Cr concentration *x*_c_ = 0.25, showing an abrupt reversal of the entire sample magnetization at the switching field *H_S_*, leading to a square-shaped hysteresis loop. Figure 9b shows instead that the magnetic field induced reversal of the sample with an increased Cr content at the center, i.e., *x*_c_ = 0.28, occurs in two steps, resembling the reversal of a system made up of two independent magnetic layers with distinct switching fields [47]. By further increasing *x*_c_, the difference in between the two switching fields increases, even though the samples still exhibit full remanence after positive and negative saturation, as shown in Figure 9c,d. Interestingly, the data in Figure 9b–d show that the magnetization value after the first-step reversal equals zero. Given the specific magnetic structure, which is symmetric with respect to its center, and the distinctive nature of the switching fields, the zero net magnetization state must correspond to a stable antiparallel (AP) configuration of the top and bottom parts of the system. However, it is worth pointing out that both VSM and SQUID experimental techniques provide exclusively macroscopic magnetic information, and are therefore by themselves not suitable to gain a deeper insight into the reversal process. 

We have therefore utilized magneto-optical (MO) measurements that are able to supply information about just the top part of the sample, because the depth sensitivity of a Kerr effect (KE)-based technique is given by the information depth of visible light. In metals, this depth is about 10–20 nm and thus, corresponds to far less than half of the total magnetic thickness of our symmetrically graded structures [48]. Therefore, in order to study only the top part of the samples, MOKE measurements along the EA have been performed and are plotted in Figure 9a–d as (red) dashed lines, in direct comparison with the VSM data. It can be promptly noticed that for the entire *x*_c_ concentration range explored, the MOKE characterization revealed a single well-defined abrupt transition from positive to negative magnetization, and vice versa. Specifically, the optically measured hysteresis loop for the *x*_c_ = 0.25 sample, displayed in Figure 9a, is fully consistent with the VSM data. They both exhibit a single transition that is moreover occurring at the same magnetic field value. This means that the ferromagnetic correlation for this sample is so strong that the system switches collectively as a single magnetic layer, even in the presence of the depth compositional gradient. By taking into account an expected Curie temperature *T_C_* ≈ 420 K for a uniform Co_0.75_Cr_0.25_ alloy film [31], the ferromagnetic exchange coupling throughout the sample structure is expected to be sufficiently strong to enable a fully correlated magnetization reversal in the entire sample. On the other hand, the MOKE measurements displayed in Figure 9b–d are partially matching the VSM data, with the latter showing an additional step in the reversal process that is not detected optically. Indeed the MOKE measurements reveal that the surface single-step transition corresponds to the second switching process in the VSM loops, meaning that the top part of each of these samples switches at higher field values than the bottom part. It is reasonable to assume that the two different switching fields for these nominally center-symmetric structures are a direct consequence of different strain fields within the top and bottom parts of the layer sequence shown in Figure 3a, which are produced in succession during the deposition process and thus grown on different effective template layers.

**Figure 10:**
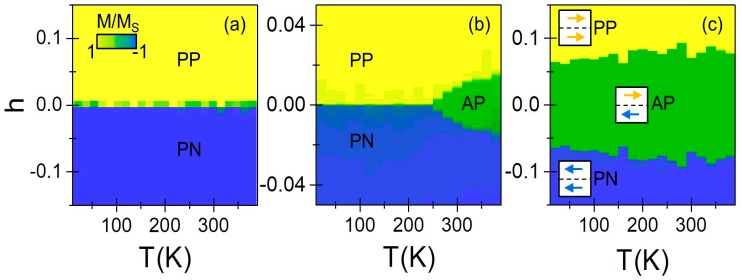
(Color online) Field-dependent magnetization as a function of temperature measured with the external magnetic field aligned along the EA for the hysteresis loop branches with decreasing field strengths for *x*_c_ = 0.25 (**a**), 0.28 (**b**), and 0.30 (**c**). The data are plotted as color-coded maps and are normalized to the saturation magnetization *M_S_* value, i.e., *M/M_S_* equal to 1 represents positive magnetic saturation PP (yellow color), −1 indicates negative saturation PN (blue color), while 0 illustrates the antiparallel case AP (green color). The insets in (**c**) illustrate the magnetic configurations of top and bottom part of the symmetric graded structure.

The combination of VSM and MOKE experimental techniques allowed us to determine that for sufficiently high *x*_c_, our samples are not anymore magnetically correlated throughout the entire thickness, and instead trigger an independent switch of bottom and top regions of the structure. The loss of ferromagnetic coupling has to be caused by the magnetic phase change of the central Co_1−_${}_{x_{c}}$Cr${}_{x_{c}}$layer, directly connected to the increase or decrease of Cr doping. At the same time, for a fixed *x*_c_ concentration, the magnetic phase of the central sample region should be strongly dependent on temperature, which may therefore trigger a temperature dependent transition between a fully correlated and partially correlated magnetization reversal. To study this, temperature dependent measurements of the magnetization reversal along the EA have been performed by SQUID magnetometry. In order to better visualize the experimental data and especially the different switching regimes, Figure 10a–c show as color-coded maps the normalized magnetization *M/M_S_* as a function of temperature and the reduced field *h* = (*H* − ${\bar{H}}_{s}$)/${\bar{H}}_{s}$, with ${\bar{H}}_{s}$ being the average of the two switching fields. In the particular case of a single-step reversal of the magnetization, ${\bar{H}}_{s}$ coincides with the actual switching field *H_S_*. Figure 10a illustrates that the single switch behavior of the magnetization occurs in the entire temperature range explored, despite the presence of a compositional gradient in the sample. This is highlighted by the presence of only two uniformly colored areas, very consistent with the expected *T_C_* of the *x* = 0.25 central alloy layer, which is higher than the highest measured temperature here. In contrast, Figure 10c shows that the magnetization reversal for the *x*_c_ = 0.30 sample occurs in two steps at any temperature that is accessible by our experimental setup, which is made visible here by the constant presence of an intermediate magnetization state with *M/M_S_* = 0 (green color). This is compatible with the expected *T_C_* for the central layer, which is predicted to be close to 10 K [49]. The intermediate *x*_c_ = 0.28 concentration sample, however, exhibits a temperature-driven transition in between the two regimes: for *T* > 260 K the reversal occurs in two steps, whereas for *T* < 260 K a single step switching is observed. This can be clearly seen in Figure 10b where the two-steps reversal, indicated by the triangular-shaped (green) area, disappears at the “transition” temperature *T* = 260 K. It is worth noting that *x* = 0.28 uniform alloy samples have been reported to exhibit a *T_C_* of approximately 270 K [31], which is in very good agreement with the measured “transition” temperature in our magnetization reversal experiment here. Two direct conclusions can already be drawn from these experimental results: firstly, the magnetization reversal mechanism can be efficiently tuned by means of graded structures of the appropriate alloy composition, which controls the effective exchange coupling along the thickness; secondly, the transition from a collective one-step to a two-steps magnetization reversal behavior can be induced by a change in temperature in samples having appropriately designed materials gradients. 

**Figure 11:**
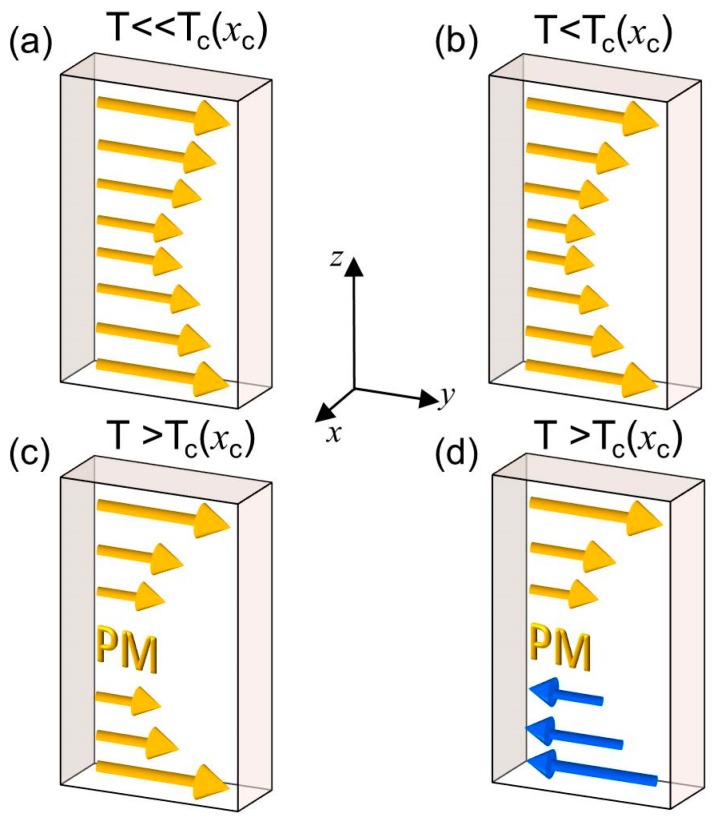
(Color online) Illustration for temperatures below (**a**,**b**) and above (**c**,**d**) the *T_C_* (*x*_c_) of the magnetization depth profile at remanence. For sufficient high temperatures, the central region becomes paramagnetic (PM) interrupting the exchange coupling throughout the structure, allowing the system to develop either a parallel (**c**) or antiparallel (**d**) configuration.

A schematic illustrating the evolution of the inferred magnetic depth profiles based on our magnetometry observations is depicted in Figure 11. The graded sample is envisioned to be made of separate exchange coupled layers, each developing a different temperature dependence of its magnetization due to different local *T_C_* values. This quantity is directly connected to the designed compositional depth profile, i.e., the lowest local *T_C_* is at the center of the structure in our sample design. If the system is at a temperature lower than *T_C_* (*x*_c_), as illustrated in Figure 11a,b, the magnetic film exhibits a continuous depth-dependent remanent magnetization profile that is following the compositional gradient. In this regime, the structure still behaves as a single magnetic system during magnetization reversal. Once the temperature crosses *T_C_* (*x*_c_), shown in Figure 11c, the structure breaks into two magnetic entities separated by an intermediate paramagnetic (PM) layer, which allows an external magnetic field to drive the structure into a meta-stable antiparallel state if the different ferromagnetic parts have distinct switching fields, as displayed in Figure 11d. It is worth mentioning that essentially the same evolution can be induced at a fixed temperature by increasing (decreasing) the maximum Cr dopant concentration *x*_c_ as we have seen in Figure 9 and Figure 10.

In order to actually confirm and not just infer the existence of these different magnetic depth structures and their field-dependent evolution, Polarized Neutron Reflectometry measurements have been performed and are presented in the next section. 

### 3.6. Polarized Neutron Reflectometry

#### 3.6.1. Magnetic Depth Profile

**Figure 12:**
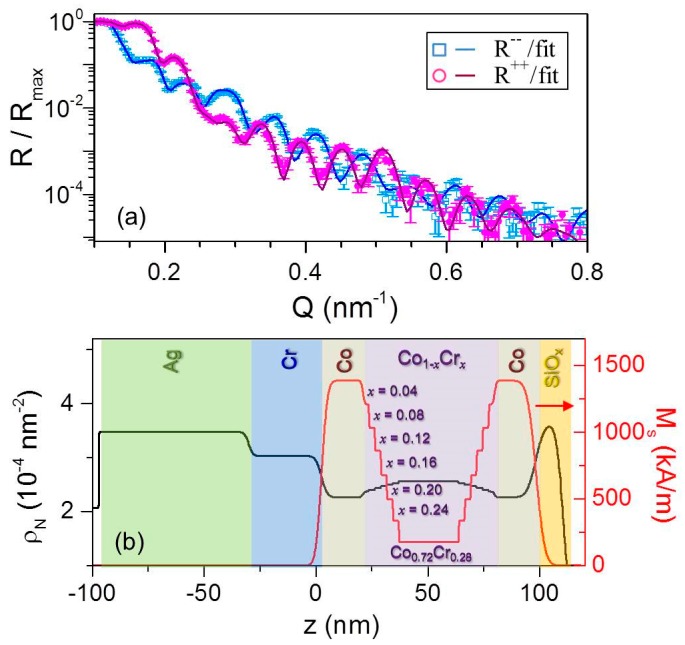
(Color online) Fitted PNR data for *x*_c_ = 0.28 measured at *T* = 4 K in *μ*_0_*H* = 1 mT along the easy axis following prior saturation via *μ*_0_*H* = 700 mT; error bars correspond to ±1 standard deviation; (**b**) scattering length density profile model, (black) straight line, and magnetization depth profile, (red) straight line, at *T* = 4 K used to fit the data in (**a**).

The characterization of the magnetic and structural depth profiles was performed by using Polarized Neutron Reflectometry (PNR). The study of the symmetrically graded structures was performed after initially saturating the samples by applying a sufficiently high positive field along the EA orientation. After this preparation step, measurements of the non-spin-flip reflectivities *R*^−^^−^ and *R*^++^ were performed as a function of the incoming beam angle in a nearly remanent state, using an applied field strength *μ*_0_*H* of only 1 mT. Hereby, it is relevant to mention that the neutron characterization was performed only on the *x*_c_ = 0.28 sample, which is the most interesting one as it exhibits a transition from one-step to two-steps reversal behavior in a temperature range that is accessible in the PNR setup. Figure 12a shows *R*^−^^−^ and *R*^++^ data measured at *T* = 4 K as a function of the transfer wave vector *Q* parallel to the surface normal. Both reflectivities show pronounced spin-dependent oscillations indicating the detailed sensitivity to both nuclear and magnetic depth gradients. The data are well fit by the nuclear scattering depth profile *ρ_N_* shown in Figure 12b as (black) straight line. It is worth mentioning that, with the exception of the Si-oxide capping layer that might be degraded, values of *ρ_N_* were fixed to expected values for all layers. This includes the graded Co_1−_*_x_*Cr*_x_* structure segments, which were modeled using a mirrored cell structure that is comprised of 7 sub-layers with *ρ_N_* corresponding to the nominal *x* composition (Figure 12b). 

The associated magnetic profile that was used to fit the PNR data is shown in Figure 12b. It is assumed to be spatially symmetric with respect to the center of the graded structure, with the Co magnetization and the magnetizations of four intermediate Co_1−_*_x_*Cr*_x_* sub-layer values being utilized as free parameters, namely the ones for the profile segments with *x* = 0.04*, x* = 0.12*, x* = 0.20, and *x* = 0.28. The intermediate sub-layer magnetization values were constrained to being equal to the average of the neighboring layer magnetizations to limit the number of free fitting parameters. The fits corresponding to the profiles displayed in panel (b) are shown as solid lines in panel (a). The fact that such a restricted model fits the data so well indicates the appropriateness of the model, which in turn corroborates that the intended compositional and magnetization depth structures were indeed both achieved. 

**Figure 13:**
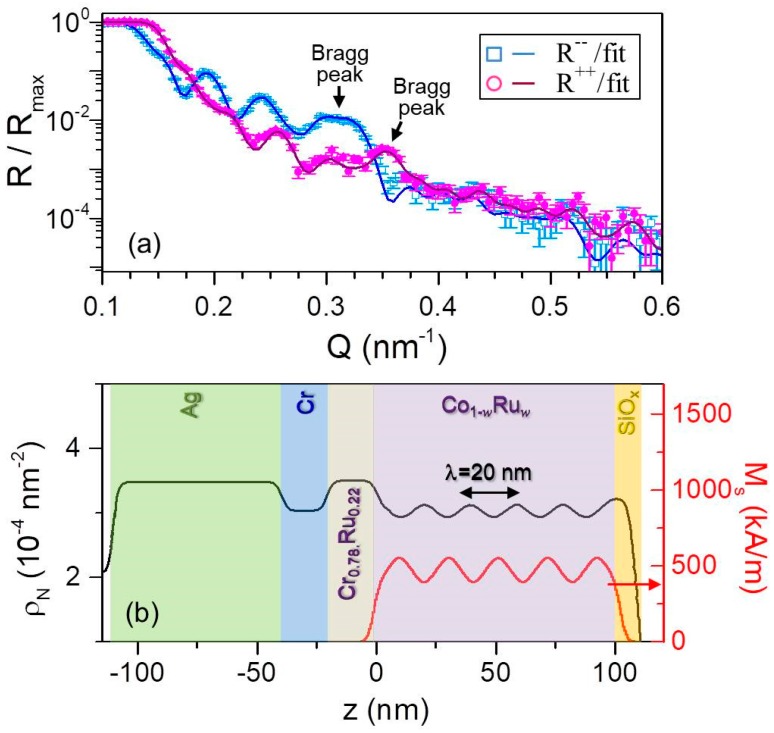
(Color online) Fitted PNR data for *λ* = 20 nm measured at *T* = 50 K in *μ*_0_*H* = 500 mT along the easy axis; error bars correspond to ±1 standard deviation; (**b**) scattering length density profile model, (black) straight line, and magnetization depth profile, (red) straight line, at *T* = 50 K used to fit the data in (**a**).

**Figure 14:**
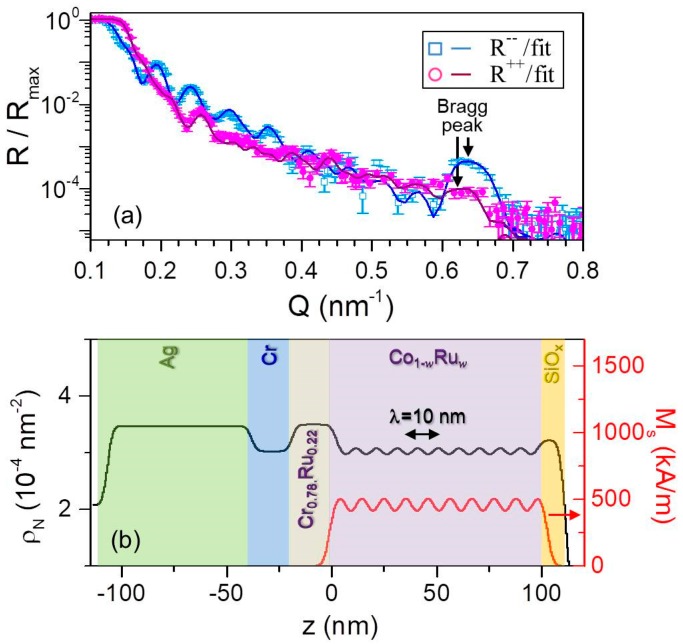
(Color online) Fitted PNR data for *λ* = 10 nm measured at *T* = 50 K in *μ*_0_*H* = 500 mT along the easy axis; error bars correspond to ±1 standard deviation. (**b**) Scattering length density profile model, (black) straight line, and magnetization depth profile, (red) straight line, at *T* = 50 K used to fit the data in (**a**).

An appropriately adapted model based on the more complex structure has been employed in order to determine whether the oscillatory magnetic modulation was achieved in the Co_1−*w*_Ru*_w_* sample structures investigated in this work. In this case, the PNR characterization was carried out in the presence of an in-plane saturating field *μ*_0_*H* = 500 mT in order to insure the collinear alignment of the magnetization in each sub-layer. Figure 13a displays the fitted *R*^++^ and *R*^−^^−^ reflectivities for the *λ* = 20 nm sample. The data exhibit pronounced oscillations generated by constructive and destructive interference as a function of *Q*, which indicates sensitivity to both nuclear and magnetic depth profiles. Moreover, the PNR data show multilayer Bragg peaks near *Q* = 2π / *λ*, marked by black arrows in Figure 13a, indicating the high degree of coherence in the structural and magnetic layering. Very good agreement was found in between the data and the fit performed by using the nuclear scattering depth profile *ρ_N_* shown in Figure 13b as (black) solid line. Just as in the case of the symmetrically graded structure, the values of *ρ_N_* were fixed to the expected values for each layer with the exception of the Si-oxide capping layer. This includes the oscillatory Co_1−_*_w_*Ru*_w_* structures, which were modeled in terms of five unit cells comprised of twenty 1 nm thick sub-layers each with *ρ_N_* corresponding to the nominal *w* composition (Figure 13b). The associated magnetic profile used to fit the PNR data is shown in Figure 13b as a (red) straight line. In order to avoid the number of free parameters becoming prohibitively large, the Co_0.69_Ru_0.__31_ and Co_0.79_Ru_0.__21_ magnetizations were utilized as fitting parameters while the intermediate magnetizations values were then assumed to vary linearly in between these two values. Also, perfect periodicity of the magnetic profile was assumed. Figure 13a shows the fitting results, as solid lines, corresponding to the profiles displayed in panels Figure 13b. Despite our heavily constrained model, both profiles result in excellent fits to the data, verifying the achievement of an oscillatory magnetic depth profile that is following the compositional one. The corresponding PNR measurement for the shorter wavelength *λ* = 10 nm sample is shown in Figure 14a. Also in this case, spin-dependent oscillations are clearly observable, indicating sensitivity to the nuclear and magnetic depth profiles. By reducing the oscillation period, the related multilayer Bragg peak shifts towards higher *Q* values, in agreement with the fact that by reducing the modulation wavelength to half its previous value the constructive diffracted signal appears at the double of the wave vector transfer. Figure 14b displays the nuclear and magnetic depth profiles utilized to fit the PNR reflectivities, based on the model above described for the *λ* = 20 nm sample. The results of the fitting are shown in Figure 14a and are in excellent agreement with the experimental data. Therefore, the PNR characterization verified that continuous compositional gradients can be used to generate continuous magnetic modulation profiles even on length scales very close to the ferromagnetic exchange length. In the remainder of our PNR result discussion, we now focus on magnetization reversal processes and its temperature dependence occurring in symmetrically graded film structures.

#### 3.6.2. Temperature Dependent Magnetization Reversal

**Figure 15:**
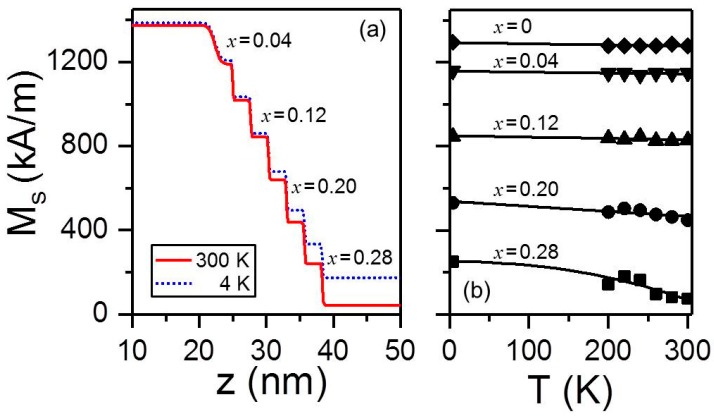
(Color online) (**a**) Magnetization depth profile used to fit the data at *T* = 4 K (red dots) and *T* = 300 K (blue dots) of the symmetrically graded *x*_c_ = 0.28 sample; (**b**) temperature dependence of the saturation magnetization for layers in the bathtub structure as determined from PNR for the same sample in (**a**). Solid curves are guides to the eyes.

Following the specific measurement protocol explained in the previous section, non-spin-flip PNR measurements were conducted over a temperature range of 4 K ≤ *T* ≤ 300 K for the *x*_c_ = 0.28 sample. Figure 15a shows the two different magnetic profiles utilized to fit the measured PNR data at *T* = 4 K (dotted blue line) and *T* = 300 K (red solid line), while the nuclear scattering profile was kept constant and is represented by the one shown in Figure 12b for all temperatures. The two profiles in Figure 15a exhibit only a small difference in the region corresponding to the outer pure Co layers, which successively increases upon increasing the dopant content, so that the central Co_0.72_Cr_0.28_ layer exhibits the largest reduction in magnetization upon increasing the temperature. The fits for other temperature values not shown here are also in very good agreement with the experimental PNR data. The magnetization values extracted from these fits for each sublayer are displayed in Figure 15b. 

**Figure 16:**
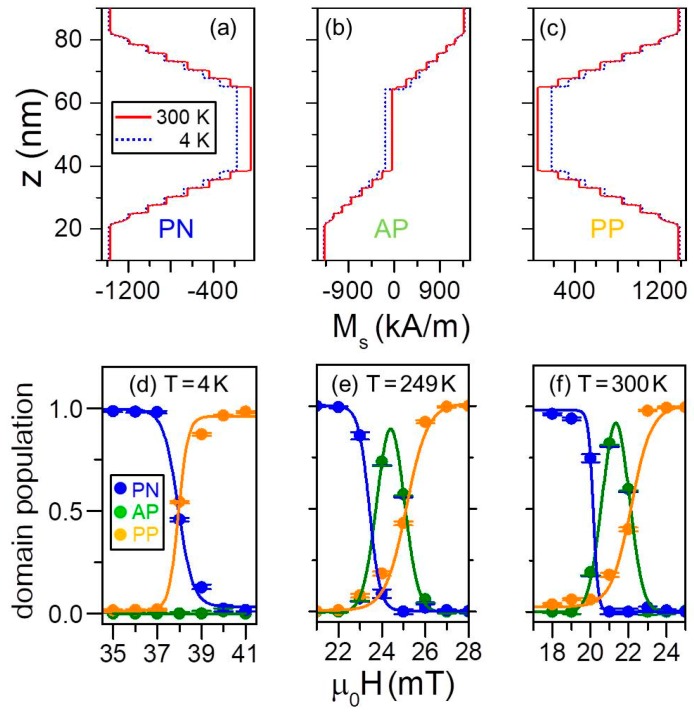
(Color online) Three magnetic depth profiles present at *T* = 4 K and *T* = 300 K used to fit PNR data measured for the sample with Cr concentration *x*_c_ = 0.28, specifically the parallel negative PN (**a**), antiparallel AP (**b**) and parallel positive PP (**c**). (**d**–**f**) field-dependent domain populations as determined from incoherent scattering model of the PNR data. Error bars correspond to ±2 standard deviations.

The Co_1−_*_x_*Cr*_x_* sublayers with 0 ≤ *x* ≤ 0.12 show a nearly temperature independent magnetization in the entire range that was measured. However, the *x* = 0.20 layer exhibits significant deviations at *T* = 200 K and above, whereas the *x*_c_ = 0.28 curve diminishes dramatically upon increasing *T*. These measurements demonstrate that the lowest local *T_C_* is located in the center of the structure, whereas the highest ordering temperature is found in the outermost regions. Therefore, the local Curie temperature is indeed changing throughout the bathtub structure, corroborating the physical picture based upon the magnetometry characterization that revealed a transition from a magnetically correlated one-step magnetization reversal at low temperature to a two-steps switching near *T* = 300 K. In order to investigate details of these temperature-dependent changes in the reversal behavior, field-dependent PNR measurements were conducted at several temperatures. At each temperature the sample was first negatively saturated by applying a field *μ*_0_*H* = −0.7 T, and subsequently PNR reflectivity data were measured for several positive field values in the range, in which magnetization switching occurs. As explained in detail in reference [27], PNR data were modeled assuming an incoherent addition of signals produced by three different magnetic configurations: parallel negative (PN) (Figure 16a), antiparallel (AP) (Figure 16b), and parallel positive (PP) (Figure 16c). At the same time, the nuclear profile was kept constant for all field or temperature values. Moreover, the sum of the probabilities to find any of the three different magnetic states was set to 1. Correspondingly, only two independent state probabilities remained as free parameters. Figure 16d–f summarize the PNR study of the magnetization reversal by displaying the field-dependent domain populations at three temperatures. Even though all three data sets reveal a non-vanishing probability of a multidomain state near the reversal for any temperature, the antiparallel configuration is irrelevant at low temperatures, but becomes most relevant as an intermediate reversal state as the temperature is increased. Thus, it has been unambiguously demonstrated that the *x*_c_ = 0.28 graded sample promotes a completely correlated magnetization reversal at *T* = 4 K, whereas the top and bottom parts of the bathtub structure reverse independently as the temperature approaches the local *T_C_* of the central layer.

## 4. Conclusions

In conclusion, we have successfully designed and fabricated epitaxial thin films exhibiting graded compositional structures by means of sputter deposition. X-ray characterization verified the epitaxial nature of our sample structures, independent from dopant concentrations and compositional depth profiles. Standard magnetometry revealed that all samples show uniaxial magnetic anisotropy, with the easy axis being parallel to the crystallographic *c*-axis in the plane of the sample. This specific magnetic anisotropy has permitted us to study the gradient profile-induced effects onto the magnetization structure in great detail. The symmetrically graded sample set disclosed a temperature- and composition-dependent magnetization reversal process. For temperatures higher than the local *T_C_* of the central layer, a two-steps magnetization reversal was observed, whereas for temperatures below that value, a fully correlated one-step reversal was measured. Specifically, samples with a Cr concentration 0.25 < *x*_c_ < 0.30 at the center exhibit a temperature-dependent transition between a one-step and a two-steps switching. In particular, we demonstrate here that a *x*_c_ = 0.28 sample undergoes this transition at *T* ≈ 260 K, triggered by the onset of ferromagnetic order of the central Co_0.72_Cr_0.28_ layer at that very temperature. 

Polarized neutron scattering measurements demonstrated the achievement of magnetic modulations that are induced by both symmetric and oscillatory graded composition depth profiles. Regarding the former sample structure, PNR experiments corroborated that the intermediate state of the two-steps reversal is indeed made up of antiparallel aligned top and bottom magnetizations. Moreover, the PNR characterization of the oscillatory composition samples revealed the possibility to continuously modulate local magnetic properties down to very short distances. Particularly, samples with Ru concentrations oscillating in between 0.21 < *w* < 0.31 with two different periods *λ* = 10 and 20 nm exhibit spatial variations of strongly and weakly magnetized regions that are caused by the nanometer scale triangular Ru concentration depth profiles. Remarkably, despite modulations on the nm length scale and the fundamentally collective nature of ferromagnetism, our experimental results are consistent with a completely localized picture of the ordered magnetic state, in which our systems behave as assemblies of uncoupled ferromagnetic layers with individual and distinct Curie temperatures [28].

## Figures and Tables

**Table 1:** *λ* dependence of the extracted *a* and *b* parameters by means of least-squares fits using Equation (1) for the data plotted in Figure 6a,b together with room temperature *M_S_* and *H_k_* values evaluated by means of least-squares fits using Equation (2) for the color-coded maps displayed in Figure 8c,d.

*λ* (nm)	*a*	*b*	*M_S_* (kAm^−1^)	*µ*_0_*H_k_* (mT)
10	0.864 ± 0.008	0.053 ± 0.004	303 ± 26	220 ± 4.3
20	0.909 ± 0.001	0.052 ± 0.004	294 ± 29	231 ± 3.2

## References

[1] Sander D., Valenzuela S.O., Makarov D., Marrows C.H., Fullerton E.E., Fischer P., McCord J., Vavassori P., Mangin S., Pirro P. (2017). The 2017 magnetism roadmap. J. Phys. D Appl. Phys..

[2] Kneller E., Hawig R. (1991). The exchange-spring magnet: A new material principle for permanent magnets. IEEE Trans. Magn..

[3] Skomski R., Coey J.M.D. (1993). Giant energy product in nanostructured two-phase magnets. Phys. Rev. B.

[4] Fullerton E.E., Jiang J.S., Grimsditch M., Sowers C.H., Bader S.D. (1998). Exchange-spring behavior in epitaxial hard-soft magnetic bilayers. Phys. Rev. B.

[5] Fullerton E.E., Jiang J.S., Bader S.D. (1999). Hard/soft magnetic heterostructures: Model exchange-spring metals. J. Magn. Magn. Mater..

[6] Fullerton E.E., Margulies D.T., Supper N., Do H., Schabes M., Berger A., Moser A. (2003). Antiferromagnetically coupled magnetic recording media. IEEE Trans. Magn..

[7] Berger A., Supper N., Ikeda Y., Lengsfield B., Moser A., Fullerton E.E. (2008). Improved media performance in optimally coupled exchange spring layer media. Appl. Phys. Lett..

[8] Fidler J., Schrefl T., Hoefinger S., Hajduga M. (2004). Recent developments in hard magnetic bulk materials. J. Phys. Condens. Matter.

[9] Wood R. (2000). The feasibility of magnetic recording at 1 terabit per square inch. IEEE Trans. Magn..

[10] Berger A. (2012). Magnetization reversal in granular thin films. Physica B.

[11] Zhou T.J., Lim B.C., Liu B. (2009). Anisotropy graded FePt-TiO_2_ nanocomposite thin films with small grain size. Appl. Phys. Lett..

[12] Kirby B.J., Watson S.M., Davies J.E., Zimanyi G.T., Liu K., Shull R.D., Borchers J.A. (2009). Direct observation of magnetic gradient in Co/Pd pressure-graded media. J. Appl. Phys..

[13] Marcellini M., Pärnaste M., Hjörvarsson B., Wolff M. (2009). Influence of the distribution of the inherent ordering temperature on the ordering in layered magnets. Phys. Rev. B.

[14] Chen J.S., Huang L.S., Hu J.F., Ju G., Chow G.M. (2010). FePt-C graded media for ultra-high density magnetic recording. J. Phys. D Appl. Phys..

[15] Kirby B.J., Davies J.E., Liu K., Watson S.M., Zimanyi G.T., Shull R.D., Kienzle P.A., Borchers J.A. (2010). Vertically graded anisotropy in Co/Pd multilayers. Phys. Rev. B.

[16] Dumas R.K., Fang Y., Kirby B.J., Zha C., Bonanni V., Nogúes J., Akerman J. (2011). Probing vertically graded anisotropy in FePtCu films. Phys. Rev. B.

[17] LeGraët C., Charlton T.R., McLaren M., Loving M., Morley S.A., Kinane C.J., Brydson R.M.D., Lewis L.H., Langridge S., Marrows C.H. (2015). Temperature controlled motion of an antiferromagnet-ferromagnet interface within a dopant-graded FeRh epilayer. APL Mater..

[18] Fert A. (2008). Nobel Lecture: Origin, development, and future od spintronics. Rev. Mod. Phys..

[19] Grünberg P.A. (2008). Nobel Lecture: From spin waves to giant magnetoresistance and beyond. Rev. Mod. Phys..

[20] Hellwig O., Berger A., Kortright J.B., Fullerton E.E. (2007). Domain structure and magnetization reversal of antiferromagnetically coupled perpendicular anisotropy films. J. Magn. Magn. Mater..

[21] Thiele J.-U., Maat S., Fullerton E.E. (2003). FeRh-FePt exchange spring films for thermally assisted magnetic recording media. Appl. Phys. Lett..

[22] Fallarino L., Berger A., Binek C. (2014). Giant temperature dependence of the spin reversal field in magnetoelectric chromia. Appl. Phys. Lett..

[23] Fallarino L., Binek C., Berger A. (2015). Boundary magnetization properties of epitaxial Cr_2-*x*_Al*_x_*O_3_ thin films. Phys. Rev. B.

[24] Victoria R.H., Shen X. (2005). Composite media for perpendicular magnetic recording. IEEE Trans. Magn..

[25] Suess D. (2006). Multilayer exchange spring media for magnetic recording. Appl. Phys. Lett..

[26] Kirby B.J., Belliveau H.F., Belyea D.D., Kienzle P.A., Grutter A.J., Riego P., Berger A., Miller C.W. (2016). Spatial evolution of the ferromagnetic phase transition in an exchange graded film. Phys. Rev. Lett..

[27] Fallarino L., Kirby B.J., Pancaldi M., Riego P., Balk A.L., Miller C.W., Vavassori P., Berger A. (2017). Magnetic properties of epitaxial CoCr films with depth-dependent exchange-coupling profiles. Phys. Rev. B.

[28] Kirby B.J., Fallarino L., Riego P., Maranville B.B., Miller C.W., Berger A. (2017). Nanoscale magnetic behavior localization in exchange strength modulated ferromagnets. arXiv.

[29] Grimsditch M., Fullerton E.E., Stamps R. (1997). Exchange and anisotropy effects on spin waves in epitaxial Co films. Phys. Rev. B.

[30] Idigoras O., Palomares U., Suszka A.K., Fallarino L., Berger A. (2013). Magnetic properties of room temperature grown epitaxial Co_1−*x*_Ru*_x_* alloy films. Appl. Phys. Lett..

[31] Inaba N., Futamoto M., Nakamura A. (1998). Temperature dependence of magnetocrystalline anisotropy energy determined using Co-Cr-Ta single crystal thin films. IEEE Trans. Magn..

[32] Inaba N., Uesaka Y., Futamoto M. (2000). Compositional and temperature dependence of basic properties of CoCr-alloy thin films. IEEE Trans. Magn..

[33] Anderson I.S., Brown P.J., Carpenter J.M., der G.L., Pynn R., Rowe J.M., Scharpf O., Sears V.F., Willish B.T.M. (2006). International Tables for Crystallography.

[34] Qiu Z.Q., Bader S.D. (2000). Surface magneto-optic Kerr effect. Rev. Sci. Instrum..

[35] Vavassori P. (2000). Polarization modulation technique for magneto-optical quantitative vector magnetometry. Appl. Phys. Lett..

[36] Felcher G.P. (1999). Polarized neutron reflectometry- a historical perspective. Physica B.

[37] Kirby B.J., Kienzle P.A., Maranville B.B., Berk N.F., Krycka J., Heinrich F., Majkrzak C.F. (2012). Phase-sensitive specular neutron reflectometry for imaging the nanometer scale composition depth profile of thin-film materials. Curr. Opin. Colloid Interface Sci..

[38] Vrugt J.A., Braak C.J.F.T., Diks C.G.H., Higdon D., Robinson B.A., Hyman J.M. (2009). Accelerating Markov chain Monte Carlo simulation by differential evolution with self-adaptive randomized subspace sampling. Int. J. Nonlinear Sci. Numer. Simul..

[39] Maeda Y., Takei K., Rogers D.J. (1993). Detection of compositional separation in Co-Ru alloy magnetic films. Jpn. J. Appl. Phys..

[40] Maeda Y., Takei K., Rogers D.J. (1994). Compositional microstructures in Co-Cr films for magnetic recording. J. Magn. Magn. Mat..

[41] Ringelstein V.P.N., Michel A., Boukari S., Bouzidi L., Persat N., Beaurepaire E., Hehn M., Muller D., Cadeville M.C. (1997). Observation of an ordered new compound Co_1−*x*_Ru*_x_* prepared by MBE on a Ru buffer layer. J. Magn. Magn. Mater..

[42] Bouzidi L., Pierron-Bohnes V., Haemmerlé O., Bouillet-Ulhaq C., Cadeville M.C. (1998). Low range chemical order and induced lattice deformation along the growth direction in epitaxial [0001] Co_1−*x*_Ru*_x_* alloys. Thin Solid Films.

[43] Ersen O., Bouzid L., Pierron-Bohnes V., Cadeville M.C. (1998). Chemical ordering along the growth direction in epitaxial [0002] Co_1−*x*_Ru*_x_* alloy thin films. Mater. Res. Soc. Symp. Proc..

[44] Idigoras O., Suszka A.K., Vavassori P., Obry B., Hillebrands B., Landeros P., Berger A. (2014). Magnetization reversal of in-plane Co films and its dependence on epitaxial alignment. J. Appl. Phys..

[45] Sun A.-C., Hsu J.-H., Sheng C.H., Kuo P.C., Huang H.L. (2007). Grain size reduction by doping Cr underlayer with Ru for longitudinal magnetic recording media. IEEE Trans. Magn..

[46] Cullity B.D., Graham C.D. (2009). Introduction to Magnetic Materials.

[47] Yuasa S., Nagahama T., Fukushima A., Suzuki Y., Ando K. (2004). Giant room-temperature magnetoresistance in single-crystal Fe/MgO/Fe magnetic tunnel junctions. Nat. Mater..

[48] Schäfer R. (1995). Magneto-optical domain studies in coupled magnetic multilayers. J. Magn. Magn. Mater..

[49] Bolzoni F., Leccabue L., Panizzieri R., Pareti L. (1983). Magnetic properties and anisotropy of Co-Cr alloy. J. Magn. Magn. Mater..

